# Cervical Cord Neurodegeneration in Traumatic and Non-Traumatic Spinal Cord Injury

**DOI:** 10.1089/neu.2019.6694

**Published:** 2020-03-06

**Authors:** Maryam Seif, Gergely David, Eveline Huber, Kevin Vallotton, Armin Curt, Patrick Freund

**Affiliations:** ^1^ Spinal Cord Injury Center, Balgrist University Hospital, University Hospital Zurich, University of Zurich, Zurich, Switzerland.; ^2^ Department of Neurophysics, Max Planck Institute for Human Cognitive and Brain Sciences, Leipzig, Germany.; ^3^ Wellcome Trust Centre for Neuroimaging, UCL Institute of Neurology, London, United Kingdom.; ^4^ Department of Neurology, University Hospital Zurich, University of Zurich, Zurich, Switzerland.

**Keywords:** biomarker, DCM, DTI, quantitative MRI, traumatic SCI

## Abstract

This study aimed to compare macrostructural and microstructural neurodegenerative changes remote from a cervical spinal cord injury in traumatic spinal cord injury (tSCI) and degenerative cervical myelopathy (DCM) patients using quantitative magnetic resonance imaging (MRI). Twenty-nine tSCI patients, 20 mild/moderate DCM patients, and 22 healthy controls underwent a high-resolution MRI protocol at the cervical cord (C2/C3). High-resolution T2*-weighted and diffusion-weighted scans provided data to calculate tissue-specific cross-sectional areas of the spinal cord and tract-specific diffusion indices of cord white matter, respectively. Regression analysis determined associations between neurodegeneration and clinical impairment. tSCI patients showed more impairment in upper limb strength and manual dexterity when compared with DCM patients. While macrostructural MRI measures revealed a similar extent of remote cord atrophy at cervical level, microstructural measures (diffusion indices) were able to distinguish more pronounced tract-specific neurodegeneration in tSCI patients when compared with DCM patients. Tract-specific neurodegeneration was associated with upper limb impairment. Despite clinical differences between severely impaired tSCI compared with mildly affected DCM patient, extensive cord atrophy is present remotely from the focal spinal cord injury. Diffusion indices revealed greater tract-specific alterations in tSCI patients. Therefore, diffusion indices are more sensitive than macrostructural MRI measures as these are able to distinguish between traumatic and non-traumatic spinal cord injury. Neuroimaging biomarkers of cervical cord integrity hold potential as predictors of recovery and might be suitable biomarkers for interventional trials both in traumatic and non-traumatic SCI.

## Introduction

Traumatic spinal cord injury (tSCI) and non-traumatic degenerative cervical myelopathy (DCM) are conditions that arise from focal cervical damage.^1,2^ The most obvious difference between a traumatic and non-traumatic cervical myelopathy lies in the time profile of neural changes (acute onset in tSCI vs. slowly developing symptoms in DCM).^3–6^ Due to progressive impairment of gait and the increasing risk of falls, DCM patients can develop a central cord syndrome, which per definition is a tSCI.^7^ Experimental evidence suggests that tSCI and DCM share several aspects of myelopathy with a combination of alpha-motoneuron damage (lesion of the central gray),^8^ demyelination,^9–12^ and axonal damage of long projecting spinal nerve fiber tracts (white matter damage),^13,14^ as well as edema and ischemic changes.^11,15^ Both etiologies present with varying degrees of upper limb impairment^16–18^ that can be assessed by comprehensive clinical protocols sensitive to sensorimotor functions (e.g., Graded Redefined Assessment of Strength, Sensibility, and Prehension [GRASSP]).^19,20^ Although such advanced clinical assessment allows quantifying the degree of impairment, it cannot disclose the underlying pathophysiology that occurs at the microstructural level.

Quantitative magnetic resonance imaging (qMRI) shows potential to detect such specific (micro-) structural changes in the spinal cord, both in tSCI^21–24^ and in DCM patients.^25–27^ To compare the magnitude of injury-induced neurodegenerative changes in both etiologies, we applied high-resolution T2*-weighted MRI and diffusion tensor imaging (DTI) above the injury level. We hypothesized that tSCI patients should show a more pronounced pattern of neurodegenerative changes compared with DCM patients, in which the disease slowly develops over time.

## Methods

### Participants and study design

Patients with tSCI at cervical level (*n* = 29; American Spinal Injury Association Impairment Scale [AIS] A-D; mean age 47.4 ± standard deviation [SD] 19.8 years, five female) and patients with mild and moderate DCM (*n* = 20, AIS D; mean age 52.0 ± 14.5 years; six female) were enrolled in this study at the University Hospital Balgrist Zurich between July 2010 and July 2015. DCM patients were recruited at >1 year after onset of symptoms and tSCI patients were recruited at least 2 months after injury (Table 1). All tSCI patients underwent decompressive surgery before study enrollment while all DCM patients were in pre-operative phase.

**Table 1.** Demographic and Clinical Information of the Traumatic SCI Patients

ID	Sex	Age, years	AIS grade	Neurologic injury level	Years since injury	GRASSP	UEMS	UELT	UEPP	SCIM
1	Male	29	A	C4	1.0	21	14	15	13	22
2	Female	40	A	C4	7.0	43	6	26	12	19
3	Male	25	A	C7	0.8	125	35	28	27	47
4	Male	34	A	C4	2.6	98	22	21	18	30
5	Male	66	A	C6	23.9	132	38	20	18	62
6	Male	68	A	C7	1.0	NT	50	28	28	NT
7	Female	39	B	C5	25.0	125	30	28	25	28
8	Male	50	B	C7	25.1	188	46	30	26	63
9	Male	53	B	C5	1.5	14	11	20	20	0
10	Female	32	C	C6	1.2	92	26	27	24	23
11	Male	70	C	C2	0.7	71	20	20	16	19
12	Male	31	C	C7	11.3	NT	50	28	26	57
13	Male	45	C	C4	20.6	80	22	24	15	27
14	Male	69	D	C7	0.2	206	49	26	25	87
15	Male	60	D	C3	0.3	NT	36	20	20	67
16	Female	63	D	C6	0.3	172	41	32	29	70
17	Male	67	D	C7	12.6	183	41	31	32	99
18	Male	56	D	C2	5.6	151	38	24	14	40
19	Male	43	D	C2	13.1	225	47	23	23	74
20	Male	27	D	C7	4.7	189	46	30	32	75
21	Male	33	D	C8	3.0	232	50	31	32	89
22	Male	51	D	C1	4.3	130	39	20	16	100
23	Male	48	D	C4	1.8	232	50	32	32	100
24	Male	50	D	C3	7.6	136	38	10	10	97
25	Male	44	D	C6	12.2	NT	50	27	28	100
26	Male	41	D	C8	3.3	NT	48	18	17	100
27	Male	52	D	C8	15.1	NT	50	28	28	90
28	Male	43	D	C6	4.6	NT	45	26	25	92
29	Male	44	D	C4	1.2	NT	50	28	28	NT

SCI, spinal cord injury; American Spinal Injury Association Impairment Scale, GRASSP, Graded Redefined Assessment of Strength, Sensibility and Prehension (maximum, 232 points); UEMS, Upper Extremity Motor Score (maximum, 50 points); UELT, Upper Extremity Light-Touch (maximum, 32 points); SCIM, Spinal Cord Independence Measure (maximum, 100 points).

The exclusion criteria for both groups were pregnancy, head or brain lesions associated with spinal cord injury, pre-existing neurological and medical disorders leading to functional impairments, mental disorder, or contraindications to MRI, and age <18 and >70 years.

The tSCI patients were divided in two subgroups based on the severity of impairment: AIS A&B group (i.e., motor complete) including 10 tSCI patients and AIS C&D group (i.e., motor incomplete) including 19 tSCI patients to better account for the severity of tSCI. A subset of subjects (17 tSCI patients and 20 DCM patients) included in the present study have been previously presented showing cord tissue specific changes induced by spinal cord injury in tSCI^23^ or cord myelopathy in DCM.^27^ Additionally, 22 healthy controls (mean age 41.1 ± 11.4 years, eight female) were enrolled to confirm the group difference between patients and healthy controls.^23,27^

All patients underwent comprehensive clinical protocols to assess neurologic and functional impairment. These included the International Standards for Neurological Classification of Spinal Cord Injury (ISNCSCI) protocol for motor score, light-touch, and pinprick score and completeness of injury; GRASSP (maximum 232 points) as ancillary outcome measures dedicated for the assessment of upper limb function^19^ and the Spinal Cord Independence Measure (SCIM). Additionally, all DCM patients were assessed using the modified Japanese Orthopedic Association (mJOA) scale (maximum 18 points). The outcome measures such as the ISNCSCI protocol for the upper extremity motor score (UEMS) (e.g. pyramidal dysfunction), light touch, pinprick, SCIM, and the GRASSP protocol were applied in both SCI and DCM patients to enable the comparison between these two etiologies.

The local ethics committee of Zurich, Kantonale Ethikkommission Zürich, approved the study (KEK-ZH-Nr. −2012-0343), and the study protocols were in accordance with the Declaration of Helsinki. Informed written consent was obtained from each subject before participation.

### MRI measurements

Participants were positioned head-first supine and acquisitions were conducted on a 3T MRI system (SkyraFit Siemens Healthcare, Erlangen, Germany). Radio Frequency (RF) excitation was performed using the body coil and detection was achieved using a combination of 12-channel head-coil, four-channel neck-coil, and 24-channel spine matrix. Subjects were stabilized with an MRI-compatible stifneck (Laerdal Medicals, Stavanger, Norway) to minimize motion artefact effects. As a result of motion artefacts, four patients (three tSCI patients and one DCM patients) and one control were excluded from microstructural assessment.

All participants underwent a protocol consisting of a T2*-weighted three-dimensional (3D) multi-echo sequence (multiple echo data image combination; MEDIC) and a diffusion-weighted imaging (DWI) sequence based on the reduced-field of view (FOV) single-shot spin-echo echo planar imaging above the injury and stenosis level. Macrostructural cord neurodegeneration was assessed by determining gray and white matter in the cross-sectional area (SCA) of the cervical cord using T2*-weighted MRI. The T2*-weighted images resulted in five high-resolution axial 3D volumes of the cervical cord with a resolution of 0.25 × 0.25 × 2.50 mm^3^ within 2.8 min acquisition time per volume. MRI parameters were as follows: FOV = 162 × 192 mm^2^, matrix size = 648 × 768, repetition time (TR) = 44 msec, echo time (TE) = 19 msec, flip angle α = 11°, and read-out bandwidth = 260 Hz per pixel. To quantify microstructural changes of the spinal cord at the identical level, a high-resolution DWI scan was applied with cardiac-gating (based on finger pulse oximetry) resulting in 30 diffusion-weighted images (b = 500 sec/mm^2^) and six b0-weighted images. The DWI sequence parameters were as follows: slice thickness = 5 mm with 10% inter-slice gap, 10 slices perpendicularly oriented to the spine, 5/8 Partial-Fourier Imaging in phase-encoding direction, phase oversampling = 50%, and a cardiac trigger delay = 200 msec, acquisition matrix = 176 × 40, FOV = 133 × 30 mm^2^, in-plane resolutions = 0.8 × 0.8 mm^2^, TE = 73 msec, and TR = 350 msec. The triggered DWI data were acquired in blocks of two slices per cardiac cycle. The minimal time between successive triggers was 1800 msec. Each DWI dataset was acquired with four averages resulting in 144 images within a nominal total acquisition time of 6.2 min.

### Data processing

#### Cross-sectional spinal cord area measurement

The serial longitudinal registration in SPM12 (Wellcome Trust Centre for Neuroimaging, University College London, UK) was applied to all T2*-weighted images to average the images accounting for intra-participant motion. Jim 6.0 software (Xinapse Systems, Aldwincle, UK) was used to merge the adjacent partitions resulting in 10 contiguous slices (to increase signal to noise ratio [SNR]) and to semi-automatically segment the cross-sectional cervical cord area using an active-surface model after setting a marker in the center of the cord in each of the 10 contiguous slices.^28^ The gray matter and white matter cross-sectional areas were manually segmented. The mean inter-observer and intra-observer reliability for these measures were shown to be in the range of previously reported results (less than 7%).^27,29^

#### Diffusion tensor imaging (DTI) measurement

Processing of DWI data was carried out with a modified version of the Matlab-based ACID toolbox within SPM12 optimized for the spinal cord.^30^ First, we reduced the in-plane FOV to 24 × 24 mm^2^ to include only spinal cord tissue. Next, diffusion weighted images were slice-wise linearly registered with 3 degrees of freedom–like translation in the frequency- and phase-encoding direction, scaling in the phase-encoding direction to correct for intra-participant motion and eddy-current artefacts.^31^ A diffusion tensor model was fitted to the DWI data by applying a robust tensor fitting algorithm that accounts for outlier volumes due to motion and physiologic artefacts^32^ and resulted fractional anisotropy (FA), mean diffusivity (MD), axial diffusivity (AD), and radial diffusivity (RD) DTI index maps. The DTI maps were spatially normalized to a self-constructed mean diffusivity template residing in the spinal Montreal Neurological Institute space.^33^ To further refine the accuracy of the registration, a manual slice-by-slice registration (in-plane translation and scaling) was performed. Finally, all DTI index maps were smoothed with a full width at half-maximum (FWHM) Gaussian kernel with 0.5 × 0.5 × 5 mm^3^. All images were visually inspected for artefacts, and the analysis was conducted on three slices from each modality at the same level.

### Statistical analysis

Statistical analysis of macrostructural MRI data, demographics and clinical outcome data was performed with Stata 15 (Stata- Corp LP, College Station, TX). The mean age was not statistically different between tSCI and DCM patients (Mann-Whitney U test: Z = 1.06, *p* = 0.29).

First, we assessed the morphometric differences in cord area, gray matter area, and white matter area between tSCI subgroups and DCM patients by means of analysis of covariance, adjusted for age. For assessing microstructural differences between patient's groups, we used voxel-based analysis of the different DTI indexes (FA, AD, RD) in SPM12, adjusted for age. All statistical parametric maps were initially thresholded with a cluster-defining threshold of *p* < 0.01 (uncorrected) and clusters surpassing a cluster threshold of *p* < 0.05 (family-wise error corrected) are reported. Next, we used linear regression analysis to investigate the relationship between cord macrostructural and microstructural changes and clinical outcome, adjusted for age. The level of significance was set to *p* < 0.05.

#### Data availability statement

Anonymized grouped data, study protocols, and processing pipelines will be shared by request from a qualified investigator.

## Results

### Clinical measures

Of 29 tSCI patients, seven were complete (AIS A) and 22 incomplete (AIS B-D). The average upper-extremity light-touch (maximum, 32), upper-extremity pin-prick (maximum, 32), and upper-extremity motor scores (maximum, 50) were (mean ± SD) 24.86 ± 5.36, 22.72 ± 6.66, and 37.52 ± 13.21, respectively. The SCIM (maximum, 100) and total GRASSP (maximum, 232) were 62.1 ± 31.99 and 135.48 ± 66.44, respectively (Table 1).

In DCM patients, the upper-extremity light-touch score (mean ± SD) was 27.70 ± 4.07, upper-extremity pin-prick score was 27.30 ± 3.77, and upper-extremity motor score was 49.70 ± 0.57. The SCIM was 97.85 ± 4.04. The total GRASSP score was 220.74 ± 12.32 (Table 2). Clinical impairment was additionally assessed based on modified Japanese Orthopedic Association (mJOA) score which identified 10 patients suffering from mild (mJOA ≥15 [max. 18]), nine from moderate (mJOA = 12–14), and one from severe (mJOA <12) DCM.

**Table 2.** Demographic and Clinical Information of DCM Patients

ID	Sex	Age (years)	Stenosis level	GRASSP	mJOA	UEMS	UELT	UEPP	SCIM
1	Male	39	C3/4^*^	225	13	50	25	23	100
2	Female	53	C5/6	230	16	50	32	30	100
3	Male	72	C7/T1^*^	222	14	50	26	22	100
4	Female	37	C3/4^*^	218	14	49	30	30	99
5	Female	58	C5/6^*^	220	16	49	29	29	100
6	Male	55	C6/7	187	12	50	23	23	98
7	Female	47	C5/6^*^	232	16	50	31	31	100
8	Male	63	C4/5^*^	219	12	50	24	24	95
9	Male	74	C6/7^*^	215	14	50	32	32	88
10	Male	32	C5/6^*^	232	16	50	20	20	100
11	Male	66	C5/6	215	9	50	25	26	86
12	Male	36	C5/6^*^	195	12	48	20	25	99
13	Male	50	C5/6^*^	231	16	49	25	26	100
14	Male	51	C5/6	217	15	50	27	27	96
15	Female	66	C5/6	216	12	49	32	32	96
16	Male	69	C5/6^*^	227	17	50	32	32	100
17	Male	68	C6/7^*^	NT	17	50	32	32	100
18	Male	39	C5/6	230	16	50	32	25	100
19	Male	34	C5/6	231	14	50	30	30	100
20	Female	31	C5/6	232	16	50	27	27	100

^*^ Multi-segmental degeneration of cervical spine.

GRASSP, Graded Redefined Assessment of Strength, Sensibility and Prehension (maximum, 232 points); mJOA, modified Japanese Orthopedic Association (maximum, 18 points); UEMS, Upper Extremity Motor Score (maximum, 50 points); UELT, Upper Extremity Light-Touch (maximum, 32 points); UEPP, Upper Extremity Pin-Prick (maximum, 32 points); SCIM, Spinal Cord Independence Measure (maximum, 100 points).

**FIG. 1.**
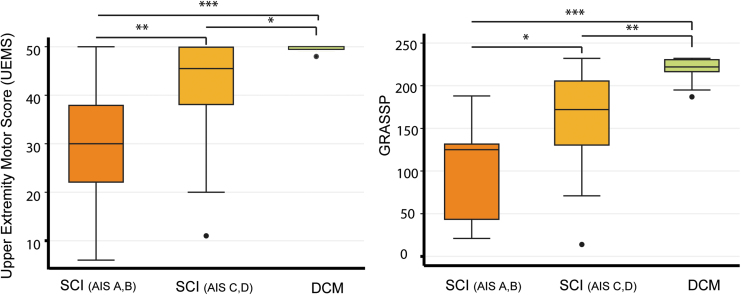
Box plots of UEMS and total GRASSP scores in spinal cord injury (SCI) and degenerative cervical myelopathy (DCM) patients. **(A)** UEMS is significantly lower in both SCI groups (AIS A&B and AIS C&D) compared with DCM patients. Additionally, UEMS of SCI with AIS A&B shows significant difference compared with SCI with AIS C&D. **(B)** GRASSP in SCI (AIS A&B and C&D groups) shows significant difference compared with the DCM patients, and there is a difference between the two SCI groups as well. UEMS, Upper Extremity Motor Scores; GRASSP, Graded Redefined Assessment of Strength, Sensation and Prehension; AIS, American Spinal Injury Association Impairment Scale. *p < 0.01; **p < 0.01; ***p < 0.001. Color image is available online.

Across group comparison, tSCI patients (divided into AIS A&B and AIS C&D) showed worse impairments in upper extremity motor score (*p* < 0.001 and *p* = 0.019, respectively) and worse GRASSP scores (*p* < 0.001, *p* < 0.001, respectively) when compared with DCM patients (Fig. 1). Pin-prick score was lower only in tSCI patients with AIS A&B compared with DCM patients (*p* = 0.008). In contrast, light-touch score was not significantly different comparing tSCI patients (AIS A&B and AIS C&D) and DCM patients (*p* = 0.32, *p* = 0.29, respectively).

### Cross-sectional spinal cord area

**FIG. 2.**
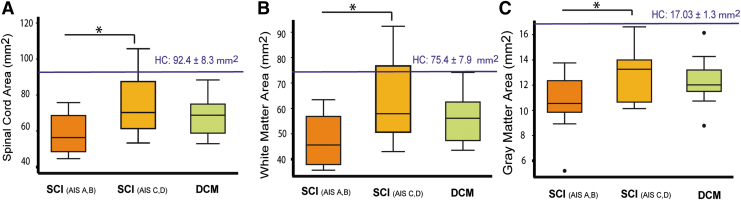
Box plots of averaged cross-sectional spinal cord, gray matter, and white matter area in spinal cord injury (SCI) and DCM patients **(A-C)** Smaller spinal cord, gray matter, and white matter area is observed in severely impaired SCI with AIS A&B grade compared with those in SCI with AIS C&D grade. However, there is no significant difference in cord atrophy comparing SCI and DCM groups. HC, healthy controls; AIS, American Spinal Injury Association Impairment Scale; DCM, degenerative cervical myelopathy. (*p < 0.01; **p < 0.01; ***p < 0.001. Color image is available online.

We first confirmed findings from previous reports that total cross-sectional spinal cord area, gray matter area, and white matter area are decreased in tSCI patients (*p* < 0.001) and in DCM patients (*p* < 0.001 ) when compared with the healthy controls.^23,27^ Between patient groups, the magnitude of remote cord atrophy (i.e., SCA) in tSCI patients (AIS A&B: 58.9 ± 11.8 mm^2^; AIS C&D: 75.3 ± 16.7 mm^2^) was not significantly different compared with DCM patients (DCM: 68.2 ± 10.4 mm^2^; AIS A&B vs. DCM: *p* = 0.37; AIS C&D vs. DCM: *p* = 0.40; Fig. 2).

Accordingly, the difference between the magnitude of atrophy in gray matter area (AIS A&B: 10.6 ± 2.6 mm^2^, AIS C&D: 12.8 ± 1.8 mm^2^, DCM: 12.4 ± 1.6 mm^2^; AIS A&B vs. DCM: *p* = 0.07; AIS C&D vs. DCM: *p* = 0.95, respectively) and in white matter area (AIS A&B: 48.3 ± 10.2 mm^2^, AIS C&D: 63.3 ± 15.3 mm^2^, DCM: 55,8 ± 9.2 mm^2^, AIS A&B vs. DCM: *p* = 0.57; AIS C&D vs. DCM: *p* = 0.22, respectively) were not significantly different when comparing tSCI to DCM patients.

### Microstructural neurodegeneration

We first confirmed by means of voxel-based analysis of the cervical cord DTI data that tSCI and DCM patients show microstructural neurodegenerative changes when compared with healthy controls.^23,27^ Specifically, we found that tSCI patients had a 16% decrease in FA (*p* < 0.0001; localization: x = 6.4, y = -19.6, z = 37; Z-score = 4.70; cluster extent = 456) and a 14% decrease in AD (*p* = 0.001; localization: x = 4.5, y = -20.0, z = 15; Z-score = 4.77; cluster extent = 234) in the dorsal columns, lateral spinothalamic, and corticospinal tract (CST) tracts when compared with healthy controls. In DCM patients, FA decreased by 18% in the lateral CST and spinothalamic tract when compared with healthy controls (*p* = 0.023; localization: x = 4.1, y = -17.0, z = 21; Z-score = 3.43; cluster extent = 105).

**FIG. 3.**
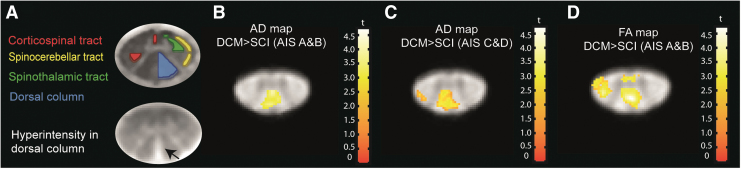
Voxel-wise analysis of microstructural changes above the level of injury (C2/C3 level) overlaid on the averaged fractional anisotropy maps across subjects in traumatic spinal cord injury (SCI) patients compared with degenerative cervical myelopathy (DCM) patients. **(A)** white matter atlas and hyperintensity signal on the T2*-weighted scan of a tSCI patient; (**B** and **C**) Decreased axial diffusivity (AD) in both SCI groups (American Spinal Injury Association Impairment Scale [AIS] A&B, *p* = 0.005; AIS C&D, *p* < 0.001) in dorsal columns and corticospinal tract compared with DCM patients. **(D)** Decreased fractional anisotropy (FA) in SCI group with AIS A&B grade compared with DCM patients in dorsal columns (*p* < 0.001). For illustration purpose, the displayed t values are uncorrected at the threshold of *p* = 0.01. Color image is available online.

Comparing tSCI with DCM patients, we found that in tSCI patients, AD was lower in the dorsal columns (AIS A&B = -14.4%; *p* = 0.005, localization: x = -0.1, y = -21.9, z = 15; Z-score = 3.39; cluster extent = 157) and AIS C&D = -12.6%, *p* < 0.001, and in the lateral corticospinal tract (AIS C&D = -11.1%, *p* = 0.041; localization: x = 1.5, y = -22.6, z = 26; Z-score = 4.33; cluster extent = 467). FA in dorsal column was lower only in severely impaired tSCI patients compared with DCM patients (AIS A&B group = -18.1%, *p* = 0.001; localization: x = 0.7, y = -21.9, z = 21; Z-score = 3.84; cluster extent = 201; Fig. 3). There were no significant differences between RD measured in tSCI and DCM patients.

### Relationship between remote neurodegeneration and clinical outcomes

**FIG. 4.**
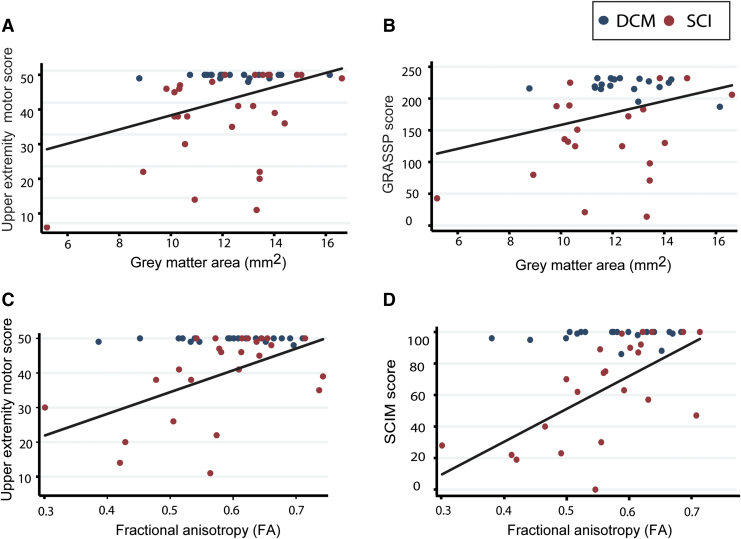
Associations between remote macrostructural and microstructural magnetic resonance imaging parameters above the level of injury (C2/C3) and clinical impairments in spinal cord injury (SCI) and degenerative cervical myelopathy patients. (**A** and **B**) the correlation between gray matter area and upper extremity motor scores (UEMS; *p* = 0.016, R^2^ = 0.2); and total Graded Redefined Assessment of Strength, Sensibility and Prehension (GRASSP) score (*p* = 0.034, R^2^ = 0.12). (**C** and **D**) Correlation between fractional anisotropy (FA) derived from the corticospinal tract and dorsal columns and UEMS (*p* = 0.008, R^2^ = 0.21) and total SCIM scores (*p* = 0.002, R^2^ = 0.27), respectively. Color image is available online.

Across all patients (tSCI and DCM), cervical cord gray matter atrophy was associated with upper extremity motor score (*p* = 0.016, R^2^ = 0.2; 95% confidence interval [CI]: 0.38–3.58, adjusted for age) and GRASSP score (*p* = 0.034, R^2^ = 0.12; 95% CI: 0.36–8.61, adjusted for age; Fig. 4). Mean FA within corticospinal tracts and dorsal columns was associated with upper extremity motor score (*p* = 0.008, R^2^ = 0.21; 95% CI. 7.38–79.30) and SCIM score (*p* = 0.002, R^2^ = 0.27; 95% CI: 66.57–232.47, adjusted for age). Microstructural and macrostructural changes in DCM patients were not significantly correlated with corresponding clinical impairments.

## Discussion

This study shows extensive cord pathology above a traumatic and non-traumatic cervical spinal cord injury. While macrostructural MRI measures revealed a similar extent of remote cord atrophy, microstructural qMRI measures were able to distinguish more pronounced tract-specific neurodegeneration in tSCI patients. The discrepancy between different clinical presentation and extensive cord pathology in tSCI and DCM patients may be suggestive of compensatory mechanisms owing to the slowly progressing disease in DCM, in contrast to the blunt and abrupt neuronal damage in tSCI patients. Our findings suggest that measures of cord atrophy are insensitive to reveal disease-specific changes while advanced qMRI measures are sensitive to the underlying disease process, as it can detect tract-specific changes.

Cervical cord atrophy remote from the injury site was previously reported in tSCI^21,23^ and DCM patients^27,34^ when compared with the healthy controls. Here, we also confirmed that the spinal cord atrophy above a cervical injury is different in both patient groups when compared with the healthy controls. However, the remote cord atrophy above the injury level is remarkably similar when tSCI patients are compared with mild DCM patients.

At the microstructural level, previous studies showed that neurodegenerative changes in remote cord regions are evident in tSCI^23,35^ and in DCM patients.^25,27,34^ In this study we show that albeit similar macrostructural cord changes, the microstructural integrity of the cord is more disturbed in tSCI when compared with DCM above the level of injury. In particular, measures of AD (indicating axonal degeneration) in the dorsal column and lateral corticospinal tract were reduced in tSCI compared with DCM. In addition, FA (indicating axonal count and myelin content)^36^ in dorsal column was significantly reduced in severe tSCI (AIS A-B) compared with DCM. Measures of AD in the dorsal column and lateral corticospinal tract and FA^36^ in dorsal column were significantly reduced comparing tSCI with DCM. Previous DTI studies in tSCI and DCM have shown increased RD and decreased FA in the supralesional cervical cord^23,27^ remote from the injury level, whereas AD values changed differently in tSCI patients when compared with DCM.^20,24^ This means that AD remote from the level of stenosis in DCM patients is increased,^27^ while it is decreased in tSCI patients. Increased AD in DCM patients may partially be due to elevated fiber tract density driven by compression and loss of surrounding cord structure,^37^ whereas in tSCI, decreased AD may be due to both axonal loss and demyelination.^23,38^

Despite the differences in etiology, the pathophysiology underlying remote cord atrophy in both tSCI and DCM patients may be driven by similar neurodegenerative mechanisms that are revealed by DTI measures. Pre-clinical studies have highlighted that a range of common primary injury mechanisms are involved in both tSCI^4^ and DCM patients,^15^ which include apoptosis of cells, inflammation, and vascular changes resulting in cell death at the focal injury site.^5,15,39,40^ Secondary injury-induced changes evolve over time and include anterograde and retrograde axonal degeneration of spinal pathyways,^5,14,41,42^ remodulation of neuronal spinal circuits,^43^ dysregulation of growth factors,^39^ shrinkage of the neuron soma size^44^ due to a reduction in muscle activity of the upper extremity, and remodeling of microvasculature configuration.^40^

Interestingly, remote neurodegenerative changes (i.e., atrophy) within the cervical gray matter in both tSCI and DCM groups are associated with upper limb motor function and strength, sensibility, and prehension of the upper limbs (i.e., GRASSP). Microstructural tract-specific changes (FA) above the level of injury also were related to measures of functional independence (i.e., SCIM) and upper limb function. These correlations, though, are mostly driven by SCI patients. Our findings are in line with previous reports showing that MRI derived measures of cord macrostructure and microstructure in the cervical cord are associated with clinical impairments.^21,23,27^ These clinicopathologic associations suggest that remote reorganizational changes, such as remodulation of intraspinal circuits,^43^ contribute to the level of upper limb function in traumatic and non-traumatic SCI. Demonstrating a link between microstructure and function by means of DTI and advanced clinical measures of upper limb function (e.g., GRASSP) points towards the applicability of such advanced qMRI measures over conventional MRI methods in clinical routines. Thus, spinal cord DTI can complement conventional MRI, with the potential to enhance current diagnosis and more importantly, predict outcome in tSCI and DCM patients. In particular, FA was found to show the strongest correlation with clinical scores (ISNCSCI scores in tSCI, mJOA and Nurick scores in DCM), where lower FA values were associated with higher impairment. Neuroimaging biomarkers sensitive to sensorimotor functions could therefore be used for the prediction of upper limb recovery and stratification for interventional trials.

This study has some limitations. DCM patients were on average 5 years older than tSCI patients. Therefore, age was considered as a covariate of no interest in all statistical analyses. Consequently, voxel-based analysis of DTI indexes in the spinal cord are still under development for the spatial normalization of the spinal cord images into a common space; an automated post-processing pipeline is a work in progress. To increase the reliability of our analysis, we therefore manually corrected the spatial normalization to the template.

## Conclusion

Despite clinical differences in traumatic and non-traumatic SCI patients, cord atrophy rostral to the level of the cervical injury is similar. However, measures of cord atrophy represent an accumulation of pathophysiological changes, and as such are insensitive to reveal disease specific changes. On the contrary, advanced qMRI measures can detect tract-specific changes that are clinically eloquent. Thus, DTI of the cervical cord might be a suitable biomarker for outcome prediction and to monitor treatment effects in interventional trials in both traumatic and non-traumatic SCI.
